# Inflammatory markers as prognostic markers in patients with head and neck squamous cell carcinoma treated with immune checkpoint inhibitors: a systematic review and meta-analysis

**DOI:** 10.3389/fonc.2024.1429559

**Published:** 2024-07-26

**Authors:** Quan Wang, Xiangzhi Yin, Shengxia Wang, Haijun Lu

**Affiliations:** ^1^ Department of Radiation Oncology, The Affiliated Hospital of Qingdao University, Qingdao, China; ^2^ Department of Orthopaedics, Affiliated Hospital of Qingdao University, Qingdao, China; ^3^ Department of Rehabilitation Traditional Chinese Medicine, Laizhou People’s Hospital, Yantai, China

**Keywords:** head and neck squamous cell carcinoma (HNSCC), PD-1, PD-L1, immunotherapy, biomarker

## Abstract

**Background:**

Various inflammatory markers, including neutrophil-to-lymphocyte ratio (NLR), monocyte-to-lymphocyte ratio (MLR), platelet-to-lymphocyte ratio (PLR), and C-reactive protein-to-albumin ratio (CAR), have been linked to the effectiveness of immunotherapy in multiple types of malignancies. We investigated how these inflammatory markers affect the prognosis of patients with head and neck squamous cell carcinoma (HNSCC) receiving immunotherapy.

**Methods:**

The databases PubMed, Embase, and Cochrane were systematically searched up until March 26, 2024, to identify relevant literature. Hazard ratios (HR) and corresponding 95% confidence intervals (CI) were extracted from the eligible studies. Data analysis was conducted using Review Manager and STATA 17.0 software to assess the impact of each indicator on prognosis. Subgroup analysis was performed to explore potential sources of heterogeneity in the data.

**Results:**

The analysis included sixteen studies with 1316 patients. A higher baseline NLR was significantly associated with poorer overall survival (OS) (pooled HR: 1.55, 95%CI: 1.14-2.11, P=0.006) and progression-free survival (PFS) (pooled HR: 1.59, 95% CI: 1.21-2.10, P<0.05). Furthermore, a high NLR after immunotherapy was strongly correlated with poor OS (pooled HR: 5.43, 95% CI: 3.63-8.12, P<0.01). Additionally, higher baseline C-reactive CAR was significantly associated with worse OS (pooled HR: 2.58, 95% CI: 1.96-3.40, P<0.01).

**Conclusion:**

The inflammatory markers NLR and CAR serve as effective prognostic biomarkers for immunotherapy in patients with HNSCC. However, the practical application of clinical detection requires further validation through large-scale prospective studies to confirm these findings and explore the underlying mechanisms.

## 1 Introduction

Head and neck squamous cell carcinoma (HNSCC) is the seventh most common cancer in the world. It is diagnosed in about 940000 people every year, and about 480000 deaths were reported in the global cancer report in 2022 (1). In America, 50% of patients are found with local advanced stage, but the 5-year survival rate of Recurrence and metastasis of head and neck squamous cell carcinoma (R/M HNSCC) is only 5% (2). Conventional chemoradiotherapy used to be the treatment modality for R/M HNSCC, but KEYNOTE-040 compares pembrolizumab with conventional platinum-based regimens and offers significant advantages (3). Immunotherapy is one of the effective treatments for R/M HNSCC. However, due to the immunosuppressive tumor microenvironment, it only plays an effect in a subset of patients with R/M HNSCC (4, 5). Scientists are looking for new ways of immunotherapy, which mainly focus on the development and combined application of immune checkpoint inhibitors, as well as the application of tumor vaccines and oncolytic viruses (6, 7). Currently, ASCO officially recommends PD-L1 as biomarker for immunotherapy in the patients with R/M HNSCC (8). At present, due to the complexity of tumor immune microenvironment(TME), the existence of tumor immune evasion, and the ununified laboratory detection technology, PD-L1 detection alone cannot find all patients with cancer who benefit from immunotherapy (9, 10). Appropriate biomarkers should be selected as a supplement to PD-L1 or combined with PD-L1 for dynamic evaluation of TME. Consequently, employing suitable biomarkers to screen patients can enhance both the rate of illness survival and the duration of survival, while simultaneously mitigating the economic burden associated with the condition.

As a major feature of tumors, inflammation drives the occurrence and progression of tumors and participates in the formation of the inflammatory tumor microenvironment, which includes inflammatory cells, hematopoietic cells, endothelial progenitor cells, fibroblasts (11–14). One of the reasons for the failure of immunotherapy in R/M HNSCC is that TME has inflammatory factors or inflammatory cells to activate inflammatory pathways, inhibit the activation of immune cells, and lead to immune resistance and tumor progression (13). Tumors will form tumor-associated inflammation in the process of tumor progression, which is related to Tumor-associated macrophages (TAM), Tregs, and tumor-associated neutrophils (15, 16). Inflammatory markers can be directly detected by peripheral blood, such as neutrophil-to-lymphocyte ratio (NLR), monocyte-to-lymphocyte ratio (MLR), platelet-to-lymphocyte ratio (PLR) and C-reactive protein-to-albumin ratio (CAR), which are related to systemic inflammation. These indicators reflect the balance between innate and adaptive immunity to guide tumor immune regulation (17). It has been confirmed that inflammatory markers are associated with poor prognosis in a variety of tumors, such as colorectal cancer, lung cancer, esophageal cancer, melanoma (18–20). The effect of NLR on the prognosis of R/M HNSCC has been comprehensively analyzed, but only limited to a certain inflammatory index (21). Previously published literature did not summarize in detail the impact of inflammatory markers on R/M HNSCC prognosis (22).

In this study, we summarized the clinical significance and prognostic value of inflammatory markers such as NLR, PLR, MLR and CAR in patients with R/M HNSCC receiving immunotherapy.

## 2 Methods

This study was conducted according to PRISMA requirements.

### 2.1 Search strategy

A literature search was conducted in PubMed, Embase, and Cochrane on March 26, 2024. The search focused on patients with head and neck squamous cell carcinoma who were treated with immunosuppressants from the establishment of the database to March 26, 2024. The search terms mainly include: (1) “Squamous Cell Carcinoma of Head and Neck” OR “Mouth Neoplasms” OR “Oropharyngeal Neoplasms” OR “Head and Neck Neoplasms OR “Laryngocarcinoma” OR “hypopharyngeal carcinoma” (2) “Programmed Cell Death 1 Receptor” OR “Immune Checkpoint Inhibitors” OR “Immunotherapy” OR “Immunotherapeutic” OR “immune therapy” OR “immune checkpoint blockade” OR “PD-L1” OR “CTLA-4” OR “avelumab” OR “nivolumab” OR “Pembrolizumab” OR “Ipilimumab” OR “Durvalumab” OR “Tremelimumab” (3) “neutrophil-to-lymphocyte ratio” OR “NLR” OR “lymphocyte-to-monocyte ratio” OR “LMR” OR “monocyte-to-lymphocyte ratio” OR “MLR” OR “platelet-to-lymphocyte ratio” OR “PLR” OR “C-reactive protein-to-albumin ratio” OR “CAR”. References of relevant literature were also read and available study data were included.

### 2.2 Selection criteria

The criteria for inclusion in this study were as follows: (1) The study focused on patients with recurrent/metastatic head and neck squamous cell carcinoma (R/M HNSCC) who received immunotherapy. (2) Before treatment, the researchers evaluated the levels of NLR (neutrophil-to-lymphocyte ratio), MLR (monocyte-to-lymphocyte ratio), PLR (platelet-to-lymphocyte ratio), and CAR (circulating tumor cell-associated ratio). (3) The study provided information on the hazard ratio (HR) and 95% confidence intervals (95% CI) for progression-free survival (PFS) or overall survival (OS) associated with NLR, MLR, PLR, or CAR.

Exclusion criteria for this study were as follows: (1) incomplete data or data could not be extracted; (2) case reports, reviews, conference articles, abstracts and animal experiments; (3) non-English language articles. If the data of the literature were repeated, the literature with complete data and high quality were included.

### 2.3 Data extraction and quality assessment

The data extraction of each study was completed independently by two researchers (WQ and YXZ), and in case of conflicts, the decision was made after consensus. The data included: year of publication, country, name of the first author, study type, number of patients, treatment regimen, study year, cut-off value, and associated HR and 95%CI for OS or PFS. HR and 95%CI were extracted from multivariate regression preferentially and from univariate regression otherwise. When HR and 95%CI were not mentioned, they were extracted from Kaplan-Meier curves by the method of Tierney et al (23).

The Newcastle-Ottawa quality assessment scale (NOS) was used to evaluate the quality of the literature, which was independently conducted by two researchers (WQ and YXZ) (24). The NOS scale score is 0-9 points. If the score≥6 points, it is a high-quality literature, otherwise it is a low-quality literature.

### 2.4 Statistical analysis

The data were analyzed by Review Manager and STATA 17.0 software. I2 was used to evaluate the size of heterogeneity of articles, and P value was used to evaluate significance, with P<0.05 being statistically significant. We utilized the fixed effect model if I2<50 or P>0.05 indicated that the study’s heterogeneity was minimal; if not, we employed the random effect model. Subgroup analysis was performed using study region, number of patients, cut-off value and treatment method to determine the source of heterogeneity. Study regions were divided into Asia and others, the number of enrolled patients was divided into <100 and ≥100, NLR cut-off value was divided into ≤5 and >5, PLR cut-off value was divided into ≤319.84 and >319.84, and the treatment was divided into nivolumab alone and nivolumab alone or without nivolumab. Finally, funnel plots and Egger’s test were used to test for publication bias.

## 3 Results

### 3.1 Characteristics of included studies

**Figure 1:**
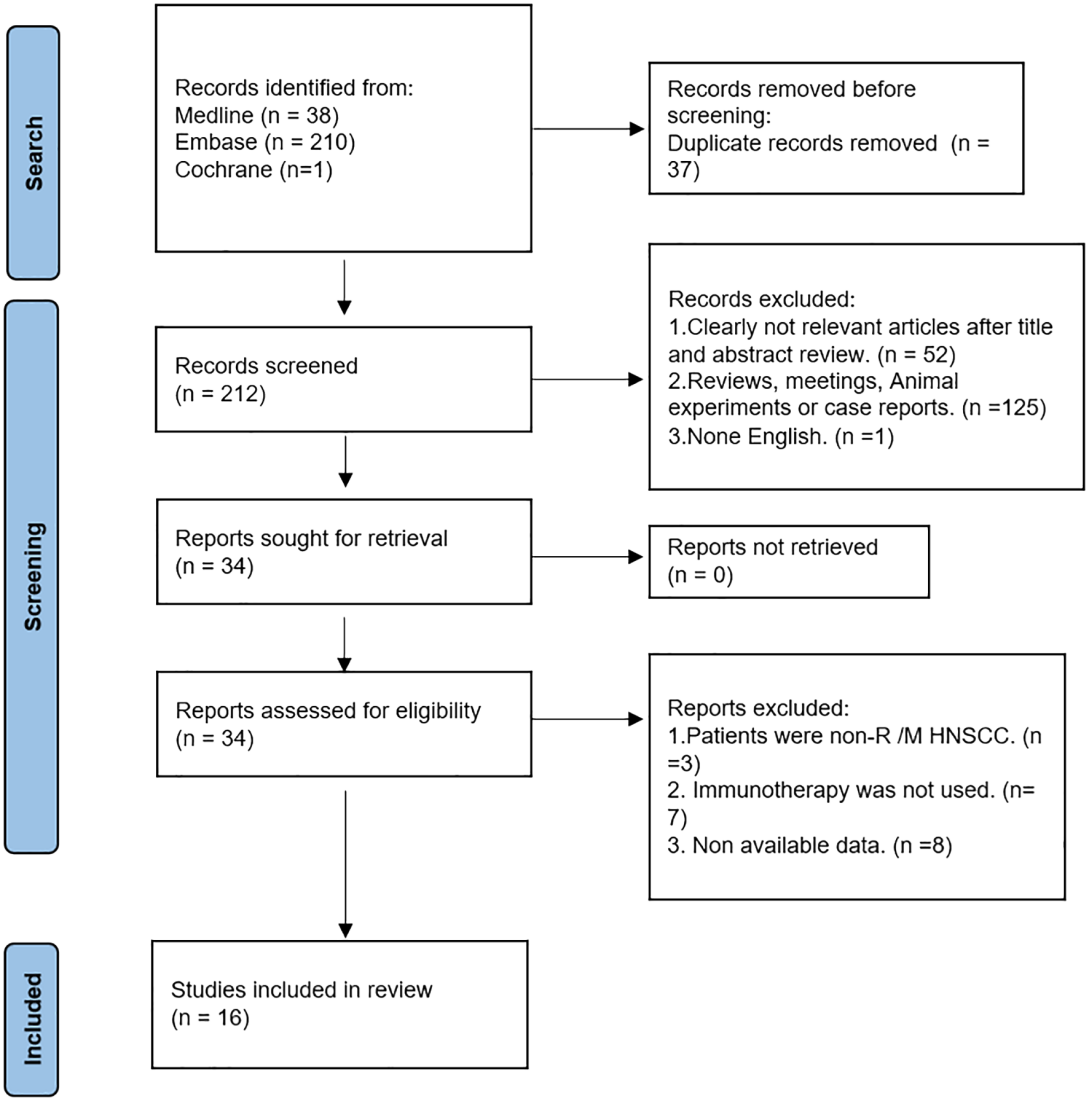
Literature search procedure for this systematic review.

For our research, we examined 249 publications obtained from the PubMed, Embase, and Cochrane databases. Out of these, we eliminated 37 duplicate papers. Through the screening of titles and abstracts, we excluded irrelevant or non-English literature, reviews, animal experiments, and conference abstracts. Next, we conducted a full-text intensive reading of the 34 articles, excluding those studying patients without R/M HNSCC, those who did not use immunotherapy, and those whose data could not be obtained. Finally, a total of 16 articles were included (25–40). The retrieval process can be seen in **Figure 1**.

The 16 studies comprised 1316 patients in total, with the number of participants varying from 14 to 164 each research. At the same time, 6 literatures were from Japan, and the rest were from Canada, China, South Korea, and the United States, etc. Except Kirsty’s study (38), which was prospective, the other 15 studies were retrospective. All the included studies included immunotherapy. (**Table 1**)

**Table 1:** Characteristics of the included studies.

Published year	Author	Country	Study design	Malignancy	Sample	Treatment	Marker	Cut-off	Outcomes	NOS
2022	MIOKO	Japan	Retrospective cohort	R/MHNSCC	164	Nivolumab	NLR	6.505	OS PFS	7
							PLR	319.84		
							CAR	0.085		
2023	Kirsty	Canada	Prospective cohort	R/MHNSCC	53	ICB	NLR	7.43	OS PFS	8
							PLR	450		
2021	Nobuyuki	Japan	Retrospective cohort	R/MHNSCC	56	nivolumab	NLR	5.2	OS PFS	8
							MLR	0.46		
							PLR	238		
2021	Pablo	London	Retrospective cohort	R/MHNSCC	99	ICI	NLR	4	OS PFS	8
2020	Takashi	Japan	Retrospective cohort	R/MHNSCC	88	Nivolumab	NLR	5.4	OS PFS	7
2022	Li	China	Retrospective cohort	R/MHNSCC	45	nivolumab or pembrolizumab	PLR	232	OS PFS	8
							NLR	5.4		
2021	Lee	Korea	Retrospective cohort	R/MHNSCC	125	ICIs	NLR	4	OS PFS	7
2023	Markus	Austria	Retrospective cohort	R/MHNSCC	79	pembrolizumab	NLR	6	PFS	7
2023	Hidetake	Japan	Retrospective cohort	OSCC	64	nivolumab	NLR	5	OS	8
2021	Kenro	Japan	Retrospective cohort	R/MHNSCC	46	nivolumab	CAR	0.3	OS PFS	7
							NLR	5		
2022	Anna	The Netherlands	Retrospective cohort	R/MHNSCC	98	PD-1 or PD-L1	NLR	4.3	OS PFS	7
							PLR	241.9		
2023	Alberto	Spain	Retrospective cohort	R/MHNSCC	100	ICIs	NLR	3	OS PFS	8
2018	Ho-1	USA	Retrospective cohort	R/MHNSCC	34	nivolumab or pembrolizumab	NLR	7	PFS	7
2018	Ho-2	USA	Retrospective cohort	R/MHNSCC	14	Other checkpoint inhibitors or combination regimens	NLR	7	PFS	7
2019	Yasumatsu	Japan	Retrospective cohort	R/MHNSCC	41	nivolumab	NLR	5	OS PFS	8
2023	Sakai	Japan	Retrospective cohort	R/MHNSCC	102	ICIs	PLR	397	OS PFS	7
							NLR	6.7		
							LMR	1.88		
2020	PARK	USA	Retrospective cohort	R/MHNSCC	108	PD-1	NLR	6.2	PFS	8

### 3.2 Baseline NLR and prognosis

**Figure 2:**
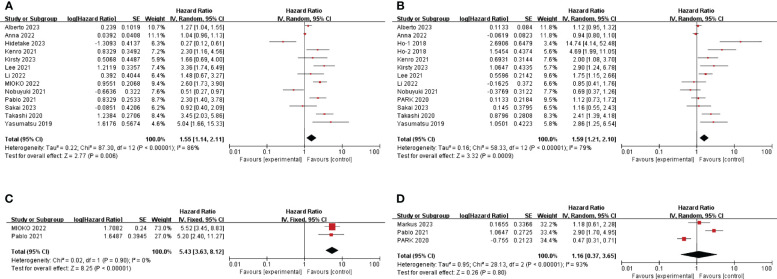
Forest plot of studies evaluating hazard ratios for the neutrophil-to-lymphocyte ratio. **(A)** Baseline NLR and OS **(B)** Baseline NLR and PFS **(C)** Post-treatment NLR and OS **(D)** Post-treatment NLR and PFS.

The HR and 95%CI of baseline NLR before immunotherapy for OS were estimated in 13 studies involving 1081 patients (25, 27, 29–33, 35–40). Considering the high heterogeneity (I^2^ = 86%, P<0.05), a model of random effects was applied. The results were a pooled HR of 1.55, 95%CI of 1.14-2.11, and P=0.006, suggesting that patients with high baseline NLR before immunotherapy had shorter OS. (**Figure 2A**) The results of subgroup analyses are shown in **Table 2**. (**Table 2**) There was no significant difference between the pooled HR and the total HR.

**Table 2:** Subgroup analysis of OS and PFS for NLR and OS for PLR.

	No of studies	HR	95%CI	P value between groups	I^2^	P value
NLR Overall survival						
Region						
Asia	9	1.56	0.87-2.82	0.617	84.1%	<0.001
others	4	1.33	1.01-1.75		76.3%	0.005
Sample size						
<100	9	1.39	0.89-2.18	0.463	82.2%	<0.001
≥100	4	1.81	1.06-3.10		82.7%	0.001
Cut-off value						
≤5	7	1.52	1.05-2.19	0.940	86.6%	<0.001
>5	6	1.47	0.81-2.7		78.2%	<0.001
Treatment						
nivolumab	6	1.52	0.65-3.52	0.945	88.6%	<0.001
non nivolumab	7	1.47	1.11-1.95		75.4%	<0.001
NLR Progression Free Survival						
Region						
Asia	7	1.49	1.02-2.18	0.737	61.2%	0.017
others	6	1.64	1.12-2.41		84.8%	<0.001
Sample size						
<100	9	1.98	1.2-3.27	0.077	84.5%	<0.001
≥100	4	1.22	1.00-1.48		22.5%	0.276
Cut-off value						
≤5	5	1.35	1.02-1.8	0.327	76.6%	0.002
>5	8	1.84	1.07-3.14		78.9%	<0.001
Treatment						
nivolumab	4	1.72	0.91-3.25	0.683	74.4%	0.008
non nivolumab	9	1.49	1.10-2.01		79.0%	<0.001
PLR Overall survival						
Region						
Asia	4	1.18	0.58-2.4	0.735	79.3%	0.002
others	1	1.43	0.60-3.41		–	–
Sample size						
<100	3	0.87	0.42-1.78	0.046	61.8%	0.073
≥100	2	1.97	1.37-2.83		0.0%	0.784
Treatment						
nivolumab	2	0.98	0.22-4.26	0.645	92.8%	<0.001
non nivolumab	3	1.41	0.88-2.26		0.0%	0.736
Cut-off value						
≤319.84	3	1.04	0.41-2.63	0.440	85.8%	0.001
>319.84	2	1.60	0.88-2.92		0.0%	0.723

The meaning of symbol “-” is that “unable to merge HR due to too few articles”.

A total of 12 articles (HO et al. ‘s study provided two cohorts) mentioned HR and 95%CI of pre-immunotherapy baseline NLR for PFS, involving 910 patients (25, 27–31, 34, 35, 37–40). After heterogeneity test (I^2^ = 79%, P <0.05), random effects model was used to analyze the data. The combined HR was 1.59, 95%CI was 1.21-2.10 and P<0.05, suggesting that patients with high baseline NLR before immunotherapy had shorter PFS. (**Figure 2B**) The results of subgroup analyses are shown in **Table 2**. (**Table 2**) In terms of sample size, the combined HR was different with the number of patients. In the ≥100 group, heterogeneity was significantly reduced, the pooled HR was smaller, and the 95%CI was even smaller. (HR=1.22, 95%CI=1.00-1.48, P=0.077) This suggests that heterogeneity may derive from the small number of patients included in the included literature.

### 3.3 NLR measured after treatment and prognosis

The correlation between NLR after immunotherapy and prognosis was analyzed. A total of 3 literatures mentioned the effect of NLR on PFS after immunotherapy (26, 33, 34). According to the heterogeneity test (I^2^ = 93%, P <0.05), a random effects model was used. The combined HR=1.16, 95%CI was 0.37-3.65, P=0.8, which was not statistically significant. (**Figure 2D**) According to the heterogeneity test, there were significant differences among the three studies. So, it is better to include more literatures to explore the relationship between NLR and prognosis after immunotherapy. The effect of NLR on OS after immunotherapy was also mentioned in 2 literatures (32, 33). The heterogeneity test showed that there was no significant heterogeneity between studies (I^2^ = 0%, P=0.90), so a fixed effect model was used. Combined HR=5.43, 95%CI was 3.63-8.12, P<0.01. This suggests that high NLR after immunotherapy is associated with short OS. (**Figure 2C**)

### 3.4 Baseline PLR and prognosis

**Figure 3:**
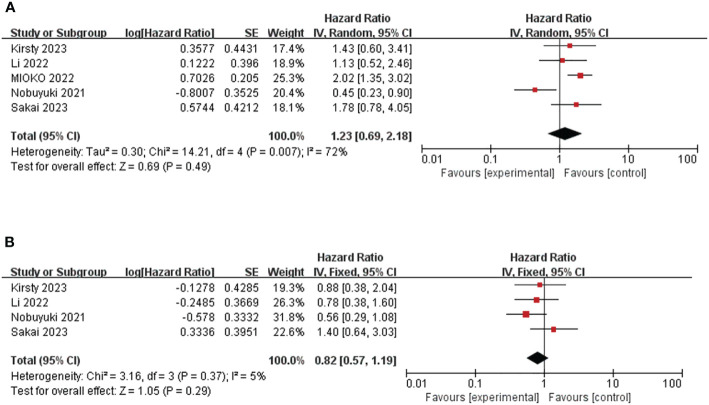
Forest plot of studies evaluating hazard ratios for the platelet-to-lymphocyte ratio. **(A)** Baseline PLR and OS **(B)** Baseline PLR and PFS.

Five studies, involving 420 patients, provided HR and 95%CI of baseline PLR before immunotherapy for OS (25, 30, 32, 35, 38). The heterogeneity test suggested that there was high heterogeneity (I^2^ = 72%, P<0.05), so the random effect model was used. The combined HR=1.23, 95%CI was 0.69-2.18, P=0.49, which was not statistically significant. (**Figure 3A**) The cut-off values of PLR >319.84 and ≤319.84 were determined according to the median cut-off value of PLR in the included literature. The results of subgroup analyses are shown in **Table 2**. (**Table 2**) The heterogeneity of the two groups regarding sample size was significantly different (I^2^ = 0.0% for the number of patients ≥100, but I^2^ = 61.8% for the number of patients <100), and the combined HR was significantly different between the two groups. The heterogeneity I^2^ was 0.0% for the group with a cut-off value >319.84. This suggests that the heterogeneity of PLR pooled HR on OS may be derived from the number of patients included in the literature and the selection of PLR cut-off values.

Four studies involving 256 patients reported HR and 95% CI of baseline PLR for PFS (25, 30, 35, 38). Heterogeneity between studies was acceptable (I^2^ = 5%, P=0.37). Through the fixed effect model, the pooled HR was 0.82, 95%CI was 0.57-1.19, P=0.29, which was not statistically significant. (**Figure 3B**) This suggests that PLR is not associated with prognosis in patients with R/M HNSCC treated with immunotherapy.

### 3.5 Baseline MLR and prognosis

**Figure 4:**
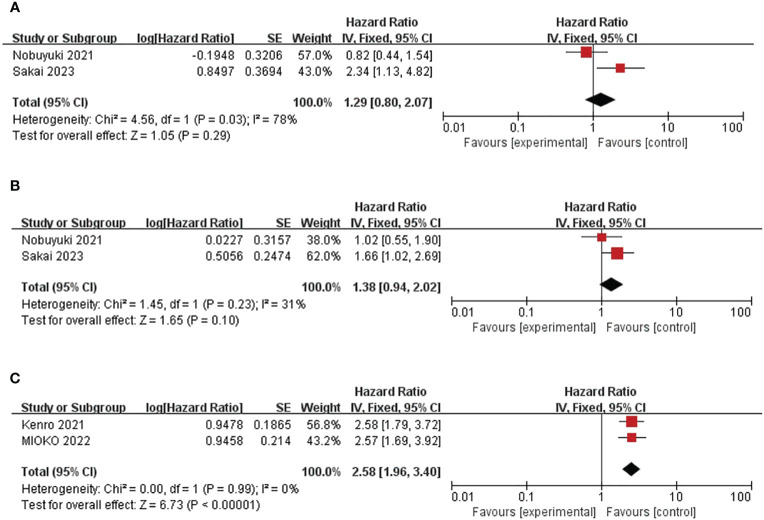
Forest plot of studies evaluating hazard ratios for the monocyte-to-lymphocyte ratio and C-reactive protein-to-albumin ratio. **(A)** Baseline MLR and OS **(B)** Baseline MLR and PFS **(C)** Baseline CAR and OS.

After literature search and screening, two studies (158 patients) showed the effect of baseline MLR on OS and PFS (25, 35). Overall, there was no correlation between the effect of MLR on OS and PFS (pooled HR for OS was 1.29, 95%CI was 0.80-2.07, P=0.29; pooled HR for PFS was 1.39, 95%CIwas 0.94-2.02, P=0.1). (**Figure 4**) Because the included studies had significant P-values, a fixed-effect model was used. The heterogeneity of OS in this study was significant (I^2^ = 78%, P=0.03). The heterogeneity of the studies on PFS was acceptable (I^2^ = 31%, P=0.23), and a random-effects model was used.

### 3.6 Baseline CAR and prognosis

In summary, two trials including a total of 210 patients showed a correlation between CAR and OS (32, 37). Combined HR was 2.58, 95%CI was 1.96-3.40, P<0.01. (**Figure 4C**) The heterogeneity test showed that the heterogeneity was acceptable (I^2^= 0%, P=0.99). This suggests that high CAR is associated with short OS in patients.

### 3.7 Quality assessment and publication bias

According to the NOS score, there were 8 articles with a score of 7 and 8. **Figure 1** lists the outcomes of all the high-quality studies that were included (**Table 1**). Publication bias was assessed using funnel plots and Egger’s test. (**Supplementary Figures 1**, **2**) Only baseline NLR for PFS had a P<0.05 Egger’s test for PFS. (P=0.008)

## 4 Discussion

Our meta-analysis, which summarized the existing literature, showed that baseline NLR and CAR of circulating inflammatory markers might predict the prognosis of patients with HNSCC receiving immunotherapy, and that higher NLR after immunotherapy is linked to a poor overall survival.

On the one hand, researchers have discovered that patients with HNSCC have a significant presence of TILs in their immunological landscape, creating a microenvironment with a high level of immune infiltration (41, 42); On the other hand, patients with HNSCC have a high tumor mutation burden (43), so immunotherapy, especially immune checkpoint inhibitors (ICIs), has significantly improved the survival time of patients with HNSCC. The selection of appropriate biomarkers will benefit more patients. Biomarkers currently recognized by ASCO are PD-L1 immunohistochemistry and Tumor mutational burden (TMB) (8). Nevertheless, PD-L1 and TMB are still not the best biological indicators considering tumor heterogeneity, laboratory detection methods (8, 10, 44, 45). At present, other prognostic markers for immunotherapy of HNSCC have been found, including tertiary lymphoid structures (TLSs) (46), Interferon-γ (IFN-γ) (47), TILs (48, 49), CTLA-4 (50). The prediction efficiency of currently known biomarkers is insufficient, so it is necessary to supplement biomarkers or find more sensitive and specific markers.

Our investigation demonstrated that elevated NLR values following immunotherapy were linked to reduced overall survival and increased pooled hazard ratio values. NLR is a combination of the interaction between inflammation and immune function. It can be considered as a sign of the balance between inflammation produced by tumor and immune function in tumor patients (19). High NLR values represent a higher density of tumor-associated neutrophils (TAN) or a lower number of lymphocytes in the peripheral blood. TAN was proved to have anti-tumor effect in cancer early. However, the patients included in our study were patients with recurrent and metastatic tumors and had advanced tumors. It has proven TAN is closely related to the tumor growth, progression, and metastasis. In the tumor microenvironment, TAN is induced to produce reactive oxygen species (ROS) and form neutrophil extracellular traps (NET), which can degrade thrombospondin-1 and promote tumor growth (51). Characteristics of suppressive T cell activity, such as PGE2 expression, were obtained with TAN (52). Due to immune escape of tumor cells, T cells are unable to exert anti-tumor effects. TAN promotes blood vessel growth and promotes tumor invasion and metastasis through various proangiogenic factors, such as VEGFA, and matrix metalloproteinase-9 (MMP-9) (53, 54). Lower lymphocytes in the peripheral blood indicate fewer TILs and are associated with poor efficacy of immunotherapy (55). This is similar to previous studies in which TME was mostly composed of exhausted T cells and the number of TILs in metastases was also relatively low. By down-regulating chemokines and immunoactive factors, cancer cells inhibit the activation of T cells and reduce the number of T cells, in order to create a tumor microenvironment of immune failure and promote metastasis (56).

In addition, Hendrik’s study demonstrated that high neutrophils were associated with a good prognosis in HPV-negative hypopharyngeal and oropharyngeal cancers (57). This may be due to tumor heterogeneity, specific neutrophil gene phenotypes, and reduced production of key components of antitumor immunity, including IL12, BATF3-dependent DCs, IFNɣ, and the CXCR3 chemokine receptor. This suggests that detecting NLR only cannot fully explain the change of the body’s immune status. It is useful that monitoring “pan-cancer” Ly6Ehi neutrophils as a biomarker for tumor immunotherapy in the future (58).

Our study showed that high NLR values after immunotherapy were associated with shorter OS and higher pooled HR values. Hwang et al. showed that the expansion of peripheral T cells induced by INF-γ resulted in a decrease in NLR and was associated with a good prognosis after immunotherapy in patients with non-small cell lung cancer (NSCLC) (19). Jeremy et al. (59) found that immunotherapy can stimulate systemic neutrophil responses in patients with cancer and demonstrated in clinical studies that patients with lung cancer with elevated NLR after immunotherapy have better prognosis than those with reduced NLR, but that is contrary to the results of our meta-analysis. Current studies suggest that there are both anti-tumor TAN and pro-tumor TAN in TME after tumor immunotherapy, and they come from different origins. The role of neutrophils after immunotherapy is related to tumor heterogeneity and the presence of cytokines that promote neutrophilic response, especially IL-2, IFN-γ (59). However, the existing studies cannot account for the mechanism of the changes in NLR kinetics in immunotherapy.

Our study suggests that high CAR values may be associated with poor OS in patients with HNSCC receiving immunotherapy. High CAR values may represent elevated CRP or decreased albumin. At present, there are few studies on the measurement of CAR values in patients with HNSCC receiving immunotherapy, which is still a relatively new field. As a major hallmark of tumors, inflammation affects the occurrence and progression of tumors by participating in the formation of inflammatory TME (60). As exogenous substances, tumor cells can induce inflammatory responses and recruit chemokines to promote the production of CRP in the liver (61, 62). Albumin reflects the nutritional status of the body (63). The low albumin level in patients with cancer is due to increased catabolism, and the cachexia state of patients with cancer leads to increased vascular permeability and increased albumin loss (64). Previous studies have shown that patients with HNSCC with lower albumin have worse prognosis (65). Therefore, CAR not only represents the level of inflammation but also the nutritional status of the body, which is a favorable prognostic indicator.

PLR and/or MLR have been found to be significant prognostic markers in malignant tumors such as NSCLC (66) and liver cancer (67, 68), but their correlation was not found in our study. The possible reasons are 1) the degree of malignancy of the tumor. We also found no significant relationship between inflammatory markers and prognosis in thyroid cancer, considering the heterogeneity of the included cancer species may affect the changes of markers (69), 2) different cells change at different times when receiving immunotherapy, considering that changes in immune cell kinetics may affect the results, 3) we performed subgroup analysis on the effect of PLR on OS. We found high PLR was associated with worse prognosis when many patients were included. A larger sample size is required for greater statistical power.

This meta-analysis demonstrated the impact of the inflammatory markers, such as NLR, MLR, CAR and PLR on the prognosis of patients with HNSCC receiving immunotherapy. We update relevant studies and firstly present the impact of CAR on HNSCC prognosis. This study is the first to analyze the prognostic impact of NLR level after immunotherapy in patients with HNSCC.

However, our study has limitations. First, most of the studies included in this study were retrospective analysis, and the funnel plot showed the existence of publication bias. Second, the number of enrolled patients may have been a source of heterogeneity based on subgroup analysis. Therefore, more and larger prospective studies are needed. Third, the cut-off values of the included literature were quite different. Finally, although the biological effects of HNSCC are similar, the prognostic changes of each cancer type are not clearly explained due to data limitations.

## 5 Conclusion

To summarize, the inflammatory markers NLR and CAR are reliable predictive indicators for immunotherapy in patients with HNSCC. Nevertheless, the implementation of clinical detection in real-world settings necessitates additional validation through extensive prospective studies to verify these findings and investigate the underlying mechanisms.

## Data availability statement

The original contributions presented in the study are included in the article/**Supplementary Material**. Further inquiries can be directed to the corresponding author.

## Author contributions

QW: Writing – original draft. XY: Writing – original draft. SW: Writing – original draft. HL: Writing – review & editing.
