# Supplementary material for: Inflammatory markers as prognostic markers in patients with head and neck squamous cell carcinoma treated with immune checkpoint inhibitors: a systematic review and meta-analysis
Source: Front Oncol. 2024 Jul 26;14:1429559. doi: 10.3389/fonc.2024.1429559 (PMC11310145; doi:10.3389/fonc.2024.1429559)
Supplement: Supplementary file 1 [file DataSheet_1.docx]

Supplementary Material

**
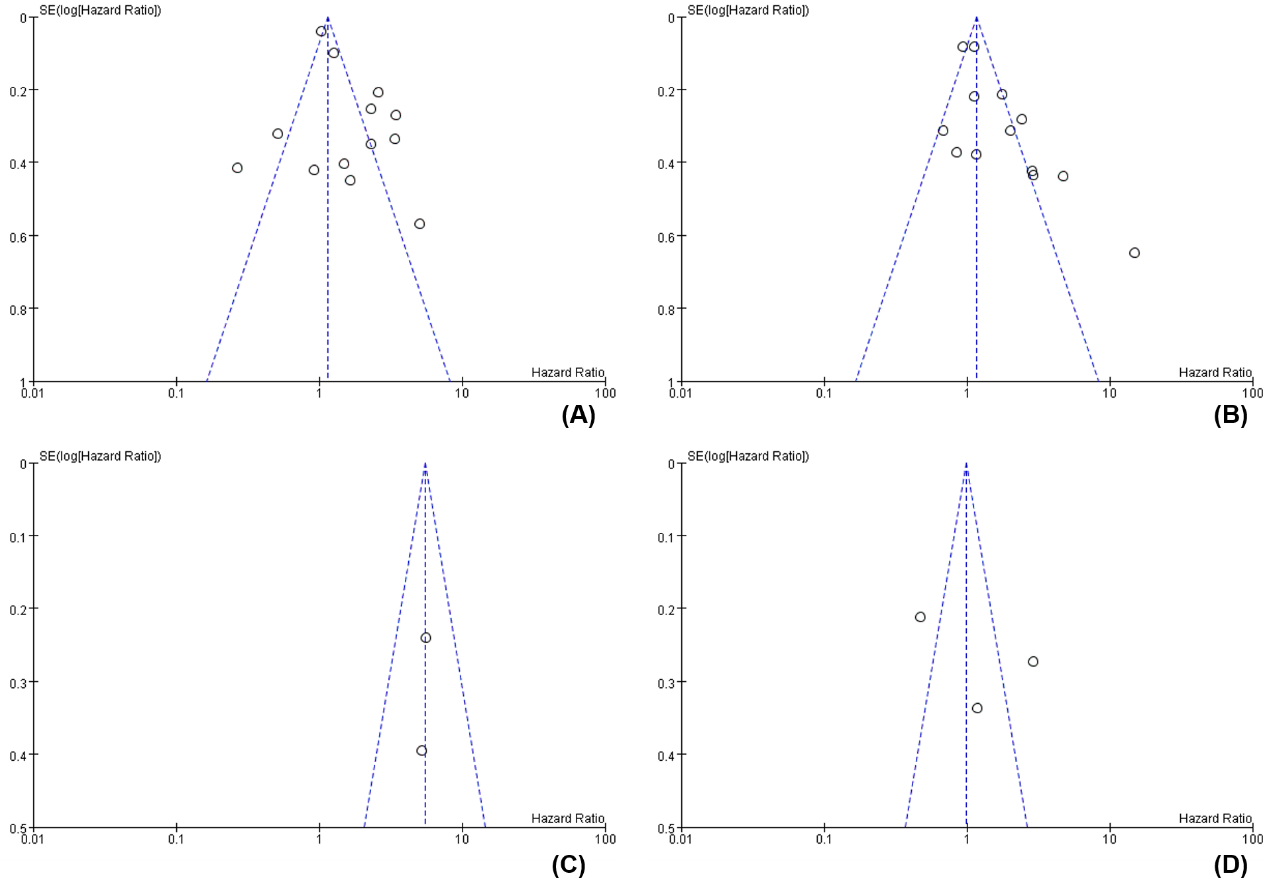
**

**Supplement Figure 1** Funnel plot of studies evaluating hazard ratios for the neutrophil-to-lymphocyte ratio. (A) Baseline NLR and OS (B) Baseline NLR and PFS (C) Post-treatment NLR and OS (D) Post-treatment NLR and PFS


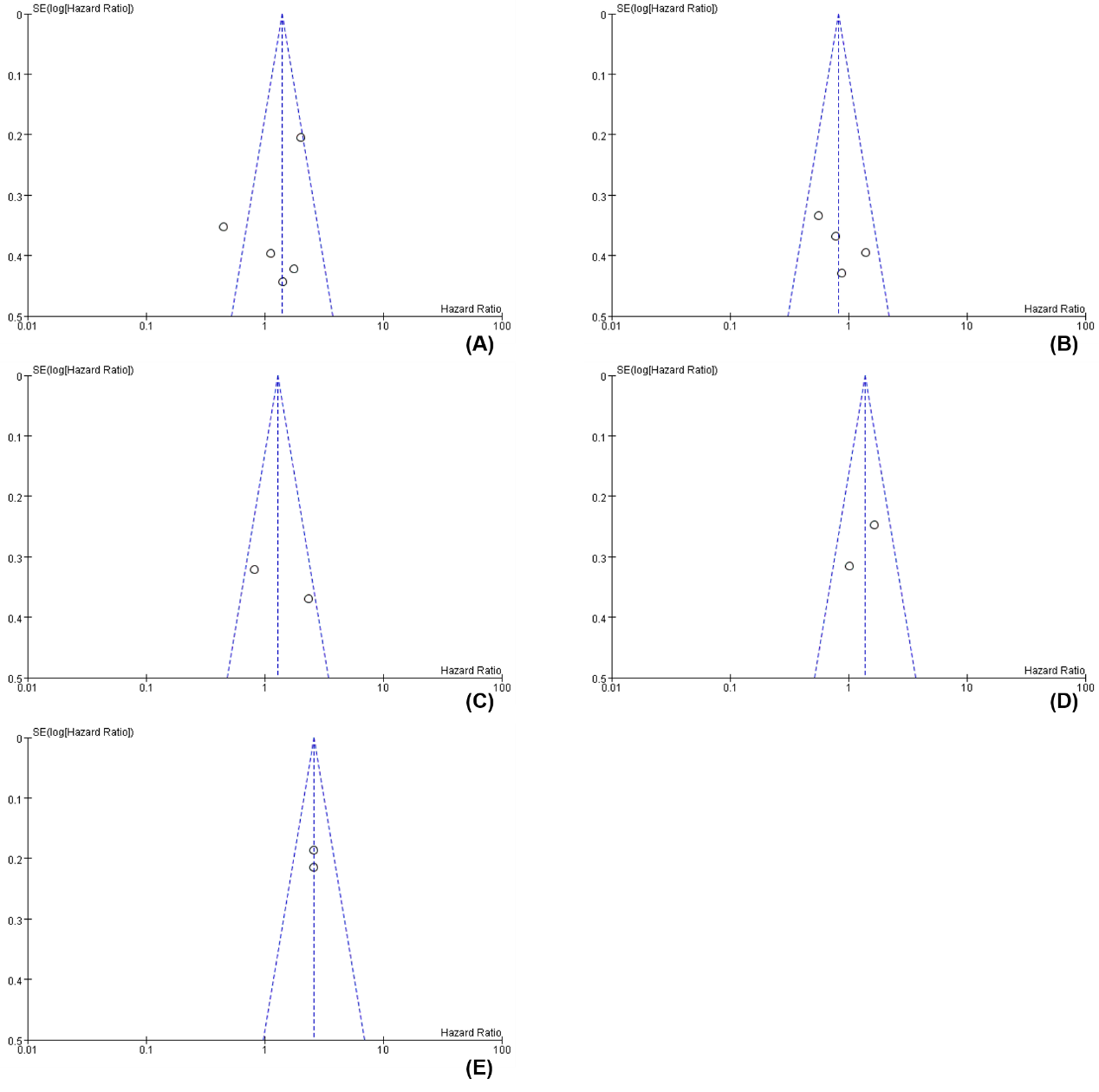


**Supplement Figure 2** Funnel plot of studies evaluating hazard ratios for the platelet-to-lymphocyte ratio, the monocyte-to-lymphocyte ratio and C-reactive protein-to-albumin ratio. (A) Baseline PLR and OS (B) Baseline PLR and PFS (C) Baseline MLR and OS (D) Baseline MLR and PFS (E) Baseline CAR and OS
